# Physical activity levels and sedentary behaviour according to sex, age, BMI, academic year, and country among medical students in Latin America

**DOI:** 10.1186/s12889-024-19133-1

**Published:** 2024-06-26

**Authors:** Diego Herreros-Irarrázabal, María Fernanda González-López, Rocío Nuche-Salgado, Josivaldo de Souza-Lima, Sandra Mahecha-Matsudo

**Affiliations:** 1 Facultad de Medicina y Ciencias de Salud, Especialidad Medicina del Deporte y La Actividad Física, Universidad Mayor, Santiago, 8580745 Chile; 2 Centro de Salud Deportiva, Clínica Santa María, Santiago, 7550000 Chile; 3 Facultad de Educación y Ciencias Sociales, Instituto del Deporte y Bienestar, Universidad Andres Bello, Las Condes, Santiago, 7550000 Chile; 4 Facultad de Educación, Universidad de Granada, Granada, 18011 Spain; 5 Centro de Investigación en Medicina Deporte, Ejercicio y Salud-Clínica MEDS, Santiago, 7550000 Chile

**Keywords:** Physical activity, Sedentary behaviour, Strength training, Medicine, Global physical activity questionnaire

## Abstract

Physical inactivity represents a global challenge in public health, being the second most significant factor contributing to mortality. In Latin America, the prevalence of physical inactivity and sedentary behaviour is notable, affecting medical students as well, who play a crucial role as behavioural role models for the population. This study addresses the prevalence of physical activity and sedentary behaviour among medical students in Latin America during the year 2023. A total of 864 participants from 12 institutions across eight countries were surveyed using the Global Physical Activity Questionnaire. Significant variations in physical activity and sedentary behaviour were observed according to sex, age, body mass index, academic year, and country. Notably, Costa Rica exhibited the highest levels of moderate physical activity in leisure time (90 min/day). Strength training was more common among men (60 min/day) and in Argentina (60 min/day). Sedentary behaviour was higher in women (420 min/day) and during the first academic year (485 min/day). Uruguay stood out with high levels of sedentary behaviour (600 min/day). Correlations indicated positive moderate associations between academic year and moderate leisure-time PA (r:0,128, p:0,007). In conclusion, there are associations between the level of physical activity and sedentary behaviour with the variables studied in this research, with the main findings being that the female sex has more time spent in sedentary behaviour (minutes/day) and less time spent in strength training (minutes/day). Additionally, there are higher levels of sedentary behaviour in the early years of medical study compared to the later years of the program.

## Introduction

The prevalence of physical inactivity (PI) stands at 31% globally, representing a current public health issue and the second most significant cause of mortality from all causes, resulting in over 5.3 million deaths [1]. PI affects various systems, being a risk factor associated with more than 35 chronic diseases [2], thereby imposing a burden and significant expenses on both public and private healthcare services [3]. Latin America is no exception in this regard. Multiple studies have demonstrated the high levels of PI present in this region. Regrettably, according to studies, Latin America ranks among the regions of the world with the highest levels of PI. In countries such as Bolivia, Chile, and Colombia, the levels of physical activity are particularly low, with prevalences of 13.9%, 13.6%, and 15.4%, respectively. Additionally, sedentary behavior is an alarming issue, with 51.8% of adolescents in Colombia reporting spending more than three hours per day sitting outside school hours [4–8].

Regrettably, according to studies, Latin America ranks among the regions of the world with the highest levels of PI [9]. Concerning sedentary behaviour (SB), the literature indicates that the average sitting time exceeds 7 h per day in some Latin American countries [5]. When evaluating the population of young adults (aged 21–30 years), they spend more time sitting during the day compared to those over 61 years of age [10]. Considering medical students, a Colombian study highlighted that the academic program with the least amount of SB was medicine when compared with seven other undergraduate courses, highlighting a weekly physical activity level of 1413 METs-min [11].

The World Health Organization (WHO) guidelines on physical activity (PA) and SB, published in 2020, stipulate that adults should accumulate at least 150 to 300 min of moderate-intensity aerobic PA, or 75 to 150 min of vigorous-intensity aerobic PA, or an equivalent combination of moderate and vigorous-intensity activities throughout the week, to attain health benefits. Furthermore, it recommends engaging in moderate-intensity muscle-strengthening activities involving major muscle groups on two or more days a week [12]. Adhering to these recommendations helps individuals achieve better levels of cardiorespiratory and muscular fitness, which have been associated with a reduction in all-cause mortality [13].

In a study conducted across several medical schools in the United States, it was observed that medical students did not meet the World Health Organization's (WHO) physical activity (PA) recommendations. Medical students engaged in significantly less moderate to vigorous physical activity (165 min per week) and spent more time in sedentary behaviour (10 h per day) compared to physical education students, who engaged in 420 min of moderate to vigorous physical activity per week and spent 7 h per day in sedentary behaviour [14]. A similar situation occurred in Brazil, where a study evaluated 186 students with an average age of 21.23 years, comparing the 2000–2001 cohort with that of 2011, and observed a significant decrease of 24.7% in compliance with the WHO's PA recommendations. SB times were 8.92 and 8.72 h/day for each group, with no significant differences between groups on weekdays [15]. On the other hand, it has been demonstrated that medical students engage in less PA than the general population [16]. Another study assessed the levels of physical activity among medical students in Colombia finding that 38.5% met the PA recommendations [17]. On the contrary, in other countries such as Argentina it was found that 79.8% of students comply with the recommendations [18]. And similar to Chile, where 77% reported a sedentary lifestyle [19] and Peru, where 25.3% of the individuals surveyed reported a low level of physical activity [20], additional research has been conducted on this topic, supporting the mentioned assertions. Moreover, it is important to highlight that scientific evidence shows that the advice given by doctors regarding healthy lifestyle habits is of higher quality and more credible to patients when the doctor practices healthy lifestyle habits themselves [21, 22]. Similarly, medical students fulfil a role akin to that of doctors.

Although this topic has been explored by various authors, to date, there has not been a study that encompasses multiple Latin American countries within the same period, providing a broader context regarding PA and SB. Therefore, the aim of this study was to compare and associate the level of PA and SB according to sex, age, body mass index (BMI), current academic year, and country of medical students in Latin America during the year 2023.

## Methods

The convenience sample was obtained following an invitation to professional contacts, student associations and/or management teams at university institutions that volunteered to participate in the study. Comprehensive information about the project and its implications was delivered via email, and the invitation was sent to 21 universities from different Latin American countries and was accepted by 12 universities from 8 Latin American countries: *Universidad de Mendoza* (Argentina), *Universidad Mayor* (Chile), *Universidad San Sebastián* (Chile), *Universidad Diego Portales* (Chile), *Universidad de La Frontera* (Chile), *Universidad de la Sabana* (Colombia), *Universidad Latina de Costa Rica* (Costa Rica), *Universidad Central de Ecuador* (Ecuador), *Universidad Americana* (Nicaragua), *Universidad Internacional* (Nicaragua), *Universidad Privada Antenor Orrego* (Peru), *Universidad de la República de Uruguay* (Uruguay). The number of medical students at these universities is approximately 19,056. To determine the necessary sample size, a sample size calculation was conducted, considering a sample universe of 19,056, with a confidence level of 95%, precision of 3% and expected proportion of 5%, the calculated sample size is 201 individuals, considering 20% expected loss, the final value is 251 individuals. Inclusion criteria were being a student from the universities, regularly enrolled, attending from the first to seventh year (notably, only Chile and Peru offer a seventh year of study in the program), and being over 18 years of age. Responses from those who did not complete and/or accept the informed consent were excluded (37 responses (4,1% of the total responses)), resulting in the final study sample of 864 reponses (comprising the 95,9% of the total of reponses). The research was conducted between August 29 and October 8, 2023. The institutional scientific ethical committee of Universidad Mayor (Folio No. 0379) approved the study, in accordance with the Declaration of Helsinki [23].

To determine the level of PA, the Global Physical Activity Questionnaire (GPAQ) version 2.0, in Spanish, proposed by the World Health Organization (WHO) and previously utilised in some Latin American countries [24], was employed. The GPAQ has been validated in the general population [25] and among university students, demonstrating high reliability [26]. This questionnaire includes 16 questions that address a typical week, focusing on the intensity (moderate and vigorous), frequency (number of days per week), and duration (minutes per day) of PA across three domains: leisure time, transport, and work. Specifically, for the work domain, considering that the participants are medical students, the GPAQ instructions advise respondents to consider time spent at work, whether in a paid position or not, or time dedicated to studying. Additionally, the questionnaire asks about the usual amount of time spent sitting or lying down on a typical day (hours: minutes per day), not counting the hours spent sleeping. Also, two questions were added to assess the frequency and duration of muscle-strengthening activities (1. During the last 7 days muscle strengthening, not including today. How many days did you carry out muscle strengthening activities?; 2. ¿How much time do you usually spend on one of those days doing physical activities for muscle strengthening?). The application of this instrument was conducted by sending a link to a Google Forms® questionnaire, with informed consent and survey to the established contacts at each university, who were responsible for distributing it to all medical students at their institution. The questionnaire included questions on sex (male/female), age (years), weight (kg), height (cm), country, years in the programme (1st, 2nd, 3rd, 4th, 5th, 6th, 7th). Body mass index (BMI Kg/m^2^) was calculated based on self-reported data and classified according to WHO classification: underweight < 18.5, normal weight 18.5—24.9, overweight 25—29.9, and obesity ≥ 30. Regarding SB, a duration of > 8 h/day was considered high [27].

In our study, the descriptive analysis of qualitative variables was presented using frequencies and percentages (%), complemented by the determination of percentiles. To ensure the consistency and quality of the collected data, the GPAQ data truncation protocol was applied. For instance, in cases where a participant reported 20 h of activity per day, this value was adjusted to 16 h/day to minimise potential errors and biases.

Quantitative variables, on the other hand, were expressed through the median and standard deviation. To assess the normal distribution of these variables, the Shapiro–Wilk test was implemented. For the comparison between variables, Student's t-test was used for parametric data and the Kruskal-Walli’s test for non-parametric data. To identify statistically significant differences between groups, the Dwass-Steel-Critchlow-Fligner test was utilized. For the correlations, the Pearson correlation coefficient was used because the data were non-parametric. A significance level of *p* < 0.05 was established for all statistical tests. All analyses were conducted using Jamovi software (version 4.3.1, 2022), thus ensuring rigorous and up-to-date data processing.

## Results

This study is based on data collected from a survey completed by 864 medical students from 12 academic institutions located in 8 Latin American countries. Table 1 displays statistical differences between men and women in health characteristics and demographic distribution of medical students in Latin America. Significantly, the Body Mass Index (BMI) averages 23.0 for women compared to 24.6 for men (*p* = 0.004), indicating that men tend to have a higher BMI. Furthermore, women predominate in all academic years, from the first to the seventh, with highly significant differences (*p* < 0.001) in each yearly comparison.

**Table 1:** Demographic distribution and health characteristics of medical students in Latin America: comparative analysis by country and academic year, 2023

		**Total (n: 864)**	**Female (n: 600)**	**Male (n: 264)**	*p*
			Mean, SD, n, %	Mean, SD, n, %	
**Age (years)**		22.0 ± 3.68	22.0 ± 3.50	22.0 ± 4.06	0.553
**BMI (kg/m**^**2**^**)**		23.5 ± 4.37	23.0 ± 4.59	24.6 ± 3.76	0.004
**Academic Year**	**1st**	84 (9.7)	60 (71.4)	24 (28.6)	< 0.001
	**2nd**	110 (12.7)	75 (68.2)	35 (31.8)	< 0.001
	**3rd**	135 (15.6)	100 (74.1)	35 (25.9)	< 0.001
	**4th**	190 (22.0)	130 (68.4)	60 (31.6)	< 0.001
	**5th**	130 (15.0)	87 (66.9)	43 (33.1)	< 0.001
	**6th**	104 (12.0)	77 (70.4)	27 (26.0)	< 0.001
	**7th**	111 (12.8)	71 (69.4)	40 (36.0)	< 0.001
**Country**	Argentina	166 (19.2)	125 (75.3)	41 (24.7)	< 0.001
	Chile	265 (30.6)	165 (62.3)	100 (37.7)	< 0.001
	Colombia	94 (10.8)	63 (67.0)	31 (33.0)	< 0.001
	Costa Rica	69 (7.9)	52 (75.4)	17 (24.6)	< 0.001
	Ecuador	92 (10.6)	56 (60.9)	36 (39.1)	< 0.001
	Nicaragua	20 (2.3)	16 (80.0)	4 (20.0)	< 0.001
	Peru	63 (7.2)	43 (68.3)	20 (31.7)	< 0.001
	Uruguay	95 (11.0)	80 (84.2)	15 (15.8)	< 0.001

*p*-values indicate the statistical significance of observed differences between men and women in each group. Statistical difference *p* < 0.005

Table 2 outlines the day minutes of PA and daily minutes SB according to sex, year of study, and country. It highlights a significant difference in moderate leisure-time PA between Chile and Costa Rica (*p* = 0.013), Colombia and Costa Rica (*p* = 0.012), and between Costa Rica and Uruguay (*p* = 0.031), with Costa Rica reporting higher levels of moderate leisure-time PA. Regarding moderate and vigorous work-related PA, a significant difference was only noted between Chile and Costa Rica (*p* = 0.003 for both). In terms of strength training, a significant difference was observed according to sex (*p* < 0.001), being higher in males, and between countries only between Argentina and Uruguay (*p* = 0.005), with Argentina showing higher levels. As for SB, the difference was significant between sexes (*p* < 0.01), being higher in females. Regarding the year of study, a significant difference in SB was observed between the first and sixth years of medical school, with SB being higher in the first year (*p* = 0.013). A similar trend was noted between the first and seventh years (*p* = 0.044). Lastly, in SB assessed between countries, there were differences between Argentina and Ecuador (*p* = 0.010), Argentina and Uruguay (*p* = 0.013), Chile and Ecuador (*p* < 0.001), Chile and Uruguay (*p* = 0.043), Colombia and Ecuador (*p* = 0.006), Ecuador and Peru (*p* = 0.031), Ecuador and Uruguay (*p* < 0.001), Nicaragua and Uruguay (*p* = 0.015), with Uruguay showing the highest values and Ecuador and Nicaragua the lowest.

**Table 2:** Comparative analysis of sedentary behaviour and physical activity by sex, and country among medical students (measured in minutes/day for SB and PA)

		**Sedentary Behaviour**			**Active Transportation**			**Moderate** **Leisure-Time PA**			**Moderate Work** **PA**			**Vigorous Work** **PA**			**Strength** **Training**		
		***Median***		***SD***	***Median***		***SD***	***Median***		***SD***	***Median***		***SD***	***Median***		***SD***	***Median***		***SD***
**Sex**	**Female**	420.0*		237.0	40.0		97.0	60.0		64.8	60.0		64.8	60.0		120.0	40.0		69.2
	**Male**	360.0		213.3	40.0		117.7	60.0		124.2	60.0		159.5	60.0		160.0	60.0*		57.1
**Country**	**Argentina**	420.0^AB^		217.0	40.0		84.4	60.0		52.4	60.0		95.9	90.0		82.4	60.0^N^		78.6
	**Chile**	420.0^CD^		214.0	35.0		88.2	60.0^I^		85.9	60.0		64.2	90.0^ M^		92.7	45.0		47.3
	**Colombia**	420.0^E^		237.0	40.0		58.3	60.0^ J^		88.7	60.0		130.2	90.0		153.0	45.0		44.7
	**Costa Rica**	360.0		233.0	60.0		87.3	90.0^IJK^		187.6	120.0		151.6	90.0^ M^		197.7	60.0		119.1
	**Ecuador**	300.0^ACEFG^		239.0	40.0		95.7	60.0		59.8	60.0		113.3	90.0		52.2	30.0		45.7
	**Nicaragua**	300.0^H^		237.0	60.0		288.4	60.0		52.5	90.0		187.1	105.0		54.8	20.0		138.9
	**Peru**	480.0^F^		238.0	50.0		136.7	60.0		65.6	60.0		1880.5	85.0		63.3	30.0		53.9
	**Uruguay**	600.0^BDEGH^		250.0	30.0		134.2	60.0^ K^		30.9	60.0		195.0	60.0		109.4	25.0^N^		47.5

*Indicates a statistically significant difference between sex (*p* < 0.001). Alphabet letters indicate significant differences between countries: (A) *p*=0.010. (B) *p*=0.013. (C) *p*<0.001. (D) *p*=0.043. (E) *p*=0.006. (F) *p*=0.031. (G) *p*<0.001. (H) *P*=0.015. (I) *p*=0.013. (J) *p*=0.012. (K) *p*=0.031. (L)  *p*=0.003. (M) *p*=0.003. (N) *p*=0.005

Regarding the correlations outlined in Table 3, it is noteworthy that weak positive correlations exist between strength training and sex (*R* = 0.072, *p* = 0.037), SB and sex (*R* = 0.139, *p* < 0.001), age and SB (*R* = -0.073, *p* = 0.037), academic year and active transport (*R* = 0.106, *p* = 0.011), and country with moderate PA at work (*R* = 0.147, *p* = 0.019). Additionally, a moderate positive correlation was observed between academic year and moderate leisure-time PA (*R* = 0.128, *p* = 0.007). Conversely, we noted a weak negative correlation for moderate and vigorous PA at work with BMI (*R* = -0.13, *p* = 0.039), and between countries with strength training (*R* = -0.082, *p* = 0.017). Lastly, a moderate negative correlation was evident for sex with moderate leisure-time PA (*R* = -0.124, *p* = 0.009), and between academic year and SB (*R* = -0.141, *p* < 0.001).

**Table 3:** Association between physical activity, sedentary behaviour, and demographic factors in Latin America medical students

		**Sedentary Behaviour**	**Active Transportation**	**Moderate Leisure-Time PA**	**Moderate Work** **PA**	**Vigorous Work PA**	**Strength** **Training**
**Sex**	R	0.139*	-0.043	-0.124	-0.048	-0.048	0.072*
	p	< 0.001	0.297	0.009	0.45	0.45	0.037
**Age**	R	-0.073	0.051	0.036	0.051	0.051	0.005
	p	0.037	0.221	0.446	0.415	0.415	0.88
**BMI**	R	0.022	-0.021	0.142	0.13	0.13*	0.046
	p	0.536	0.62	0.142	0.039	0.039	0.178
**Academic year**	R	-0.141	0.106*	0.128*	0.096	0.096	0.037
	p	< 0.001	0.011	0.007	0.125	0.125	0.288
**Country**	R	0.052	0.079	0.773	0.147*	0.147*	-0.082
	p	0.139	0.058	0.773	0.019	0.019	0.017

^*^= Statistically significant difference

**Figure 1:**
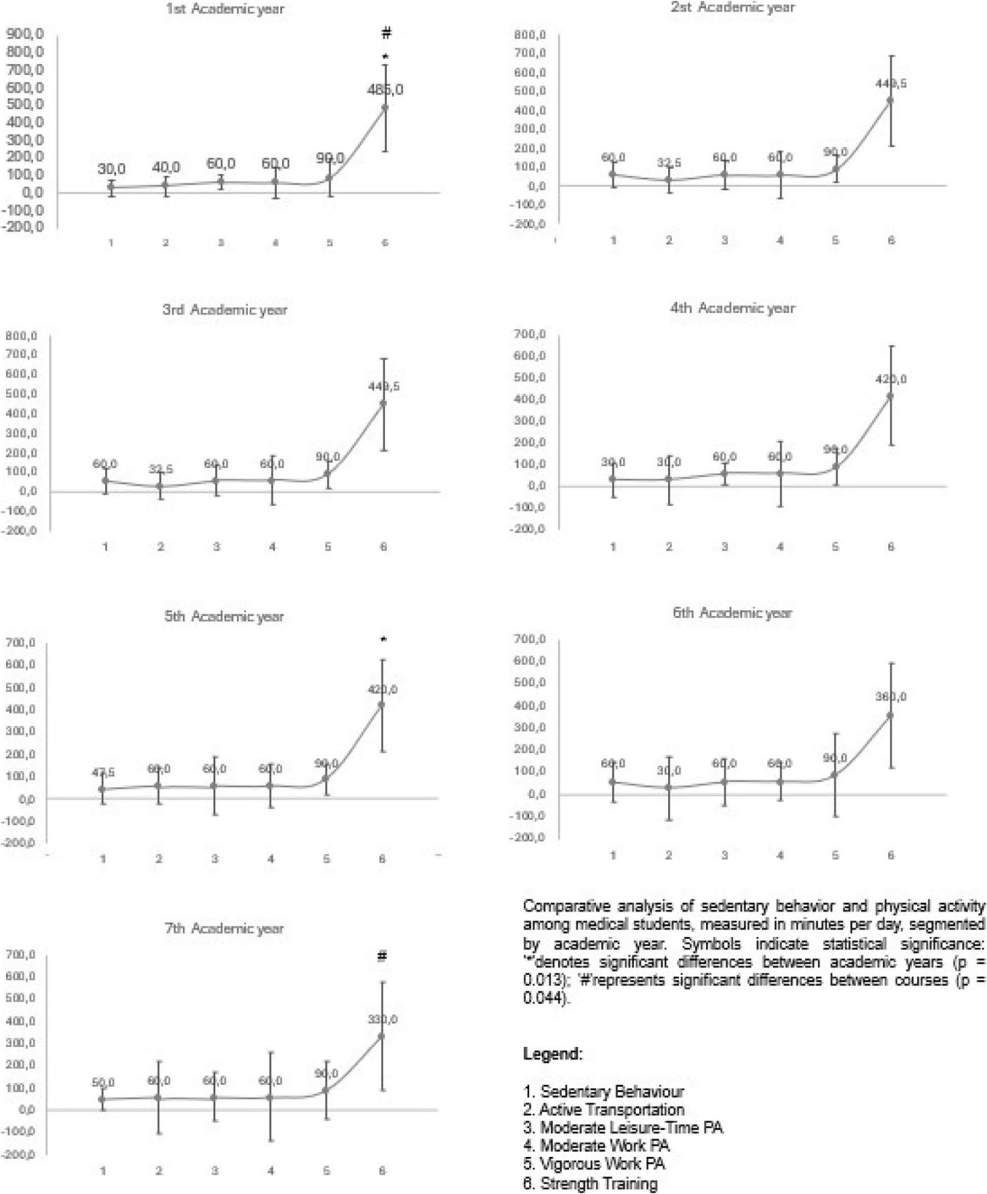
**Fig. 1**

The Fig. 1 presents a series of scatter plots displaying the trends in sedentary behaviour and various forms of physical activity among medical students across seven academic years. Each graph corresponds to a specific academic year, from the first to the seventh, illustrating median values for six categories of activity: 1) Sedentary Behaviour, 2) Active Transportation, 3) Moderate Leisure-Time Physical Activity, 4) Moderate Work Physical Activity, 5) Vigorous Work Physical Activity, and 6) Strength Training. These activities are measured in minutes per day.

## Discussion

The primary aim of this study was to compare and associate the levels of PA and SB with sex, age, BMI, current academic year, and country of medical students in Latin America during the year 2023.

64.4% of the participants in this study were women, a trend reflected in the analysis of survey responses by course and country, where a female prevalence was observed in all categories. When comparing these data with previous research, a significant difference was found, as these studies highlighted the male sex as predominant at the time of response [18, 20]. Although there are studies where a larger number of participants were female [17, 28], these had a smaller population than the present study, and none exhibited as marked a difference as this investigation. This discrepancy could be attributed to a higher number of women in the medical schools where the survey was conducted or to a higher response rate among them. According to data from the Association of American Medical Colleges (AAMC), women comprised 56.6% of applicants, 55.4% of matriculants, and 54.6% of total enrolment in U.S. medical schools for the 2023–2024 academic year, marking the fifth consecutive year that women have constituted the majority in these categories. Additionally, the American Medical Association (AMA) reported that in 2019, for the first time, women made up more than half of all medical school students, reaching 50.5% of total enrolment [29]. On the other hand, there was a significant difference in BMI averages by sex, being higher in men (24.6 ± 3.76). This finding is supported by the study of Janampa-Apaza, which showed that 50.2% of the male population was overweight or obese versus 19.9% of the female population [20]. It should be noted that BMI does not differentiate excess weight according to composition, leading to potential misinterpretations of its results. For example, an individual with a higher percentage of muscle mass might have a BMI value classifying them as overweight or obese without exhibiting the characteristics of this category. This is a variable to consider in this study, as men reported spending more time on strength exercises than women.

In terms of the outcomes within the PA domains, the resultant values were significantly lower than those documented in Argentina in 2014, employing the same questionnaire [19]. Upon evaluating the level of PA across various domains, our research indicated notable differences among countries, with Costa Rica emerging as the country with the highest weekly minutes of moderate leisure-time PA (90 min/week), moderate work-related PA (120 min/week), and vigorous work-related PA (90 min/week). This finding is particularly striking as it surpasses what has been reported in the literature concerning the general population of Costa Rica [28, 30].

Regarding SB, a notable difference was observed between sex, with women reporting higher levels than men (420 and 360 min per day, respectively), which corroborates the findings of Janapa-Apaza et al. [20], who studied a similar population at a university in Peru and noted that 50.9% of female participants spent more than 8 h per day seated. This contrasts with reports in the general Latin American population, where men reported more minutes seated (479.1 vs 442.7 min per day) [5]. In terms of academic year, there was a significant difference in SB between the first and sixth year of medical school, with higher values in the first year (485 vs 360 min per day), and a similar trend between the first and seventh year (485 vs 330 min per day) in Chile and Peru. Regarding this point, other studies have shown contradictory results, with similar findings in studies differentiating between clinical and preclinical courses [18], and differing results in another Chilean study [20]. The findings of our study suggest that the reduced SB observed in the later years, particularly in the sixth (360 min/day) and seventh year (330 min/day), can be attributed to the increased PA necessitated by clinical internships. Conversely, the early years of the program, characterized by a higher volume of theoretical classes, exhibit increased SB time (485 min/day) in comparison to the clinical courses. As mentioned in the materials and methods section, only Chile and Peru include a seventh year of study in the program, yet the previously described trend also applies to the sixth year. This should be considered when extrapolating the results.

When analysing SB by country, existing evidence aligns with our findings, showing a high level of SB across all Latin American countries [5, 28, 30]. In our study, the average SB among the countries surveyed was 360 min per day, with Ecuador and Nicaragua reporting the lowest levels of SB (300 min per day), while Uruguay reported the highest (600 min per day). It would be of interest for future research to explore the context of each of these countries in terms of public policies related to PA and the reduction of SB, to analyse these results in greater detail and generate new, high-quality information that could be used for interventions.

In our research, a weak positive correlation was described between SB and sex, indicating that women tend to exhibit slightly higher SB than men, as previously discussed in this publication. Additionally, a moderate positive correlation was observed between the academic year of the participant and the level of moderate leisure-time PA, suggesting that as students’ progress in their education, they tend to engage in more moderate PA during their leisure time. Another significant finding was the moderate negative correlation between sex and moderate leisure-time PA, interpreted as female participants being less likely to engage in moderate leisure-time PA compared to their male counterparts. Finally, there was a moderate negative correlation between academic year and SB, interpreted as individuals tending to decrease their SB as they advance in their studies. To our knowledge, this type of analysis is not described in the existing literature on the subject, though similar trends have been observed in previous studies [5, 20].

Among the strengths of this research, it is noteworthy that this is the first study to measure the level of PA and SB among medical students from eight Latin American countries, enabling the generation of more comprehensive results and, thereby, conclusions that more closely reflect the local reality. This is related to the total number of participating individuals since, in previous research, there has not been a study with a participant count like or greater than this one. Another aspect to consider is that out of the 864 individuals who participated, 95.9% completed the survey accurately, which allowed for minimal loss of information. Lastly, it is important to highlight the inclusion of an evaluation of muscle-strengthening exercise practices, which is pertinent information since muscular fitness has been recognized as a mortality risk factor, hence its inclusion in the WHO recommendations since 2020 [11].

This study has several limitations that should be considered when interpreting the findings. First, although it includes multiple Latin American countries, generalizing the results across the entire region may be limited due to unexplored variations in academic and cultural contexts. Additionally, the reliance on the GPAQ questionnaire to measure physical activity and sedentary behavior might not capture all dimensions of these behaviors, as it is based on self-reporting, which is subject to recall and social desirability biases. Lastly, the inclusion of a seventh academic year only in Peru and Chile necessitates caution when comparing academic data across all countries, as these specific program structures could influence the patterns of physical activity and sedentary behavior.

We can conclude that there are various associations between the level of PA and SB with variables such as sex, age, BMI, academic year, and the country of medical students in Latin America. Women spent more time in SB and less in strength exercises. There were higher levels of SB in first-year students compared to those in the last years.

## Data Availability

Not applicable.

## Abbreviation

PI: Physical Inactivity
SB: Sedentary Behaviour
WHO: World Health Organization
PA: Physical Activity
GPAQ: Global Physical Activity Questionnaire
BMI: Body Mass Index
